# Curved-crease origami face shields for infection control

**DOI:** 10.1371/journal.pone.0245737

**Published:** 2021-02-08

**Authors:** Aurimas Bukauskas, Antiopi Koronaki, Ting-Uei Lee, Daniel Ott, M. Wesam Al Asali, Aftab Jalia, Tom Bashford, Ana Gatóo, Josh Newman, Joseph M. Gattas, Darshil U. Shah, Michael Ramage

**Affiliations:** 1 Centre for Natural Material Innovation, Department of Architecture, University of Cambridge, Cambridge, United Kingdom; 2 School of Civil Engineering, University of Queensland, St. Lucia, Australia; 3 Department of History and Philosophy of Science, University of Cambridge, Cambridge, United Kingdom; 4 NIHR Global Health Research Group on Neurotrauma, Division of Anaesthesia, Addenbrooke’s Hospital, University of Cambridge, Cambridge, United Kingdom; 5 Healthcare Design Group, Engineering Design Centre, Department of Engineering, University of Cambridge, Cambridge, United Kingdom; 6 School of Media, University of Brighton, Brighton, United Kingdom; Northwestern University, UNITED STATES

## Abstract

The COVID-19 pandemic has created enormous global demand for personal protective equipment (PPE). Face shields are an important component of PPE for front-line workers in the context of the COVID-19 pandemic, providing protection of the face from splashes and sprays of virus-containing fluids. Existing face shield designs and manufacturing procedures may not allow for production and distribution of face shields in sufficient volume to meet global demand, particularly in Low and Middle-Income countries. This paper presents a simple, fast, and cost-effective curved-crease origami technique for transforming flat sheets of flexible plastic material into face shields for infection control. It is further shown that the design could be produced using a variety of manufacturing methods, ranging from manual techniques to high-volume die-cutting and creasing. This demonstrates the potential for the design to be applied in a variety of contexts depending on available materials, manufacturing capabilities and labour. An easily implemented and flexible physical-digital parametric design methodology for rapidly exploring and refining variations on the design is presented, potentially allowing others to adapt the design to accommodate a wide range of ergonomic and protection requirements.

## 1 Introduction

The COVID-19 pandemic has created unprecedented global demand for large quantities of Personal Protective Equipment (PPE). Face shields have been identified as an important component of PPE for frontline healthcare workers. They are classed as “adjunctive PPE”, intended to be worn in addition to respiratory protection to provide additional protection of the facial area from splashes and sprays of bodily fluids from infected patients, specifically to the mucous membranes of the eyes, nose, and mouth [1, 2].

### 1.1 Challenges in PPE provision

#### 1.1.1 PPE demand forecasting

Using the WHO COVID-19 Essential Supplies Forecasting Tool (ESFT) (version 3) [3], the global demand for face shields for the 12 week period of 22 November 2020—14 February 2021 is estimated to be approximately 1.04 billion units, corresponding to a predicted cumulative 1.3 billion cases of COVID-19 over this period. At an estimated cost of $0.60 per shield given by the ESFT, this equates to an approximately $620 million global expenditure on face shields over this period. These demand estimates were obtained assuming the default Susceptible, Infected, and Recovered (SIR) epidemiological model incorporated in the ESFT, with a cumulative global diagnosed COVID-19 case count of 58.6 million as of 22 November 2020. A global population of 7.8 billion was assumed. Default values provided by the ESFT for healthcare system equipment, diagnostic, and treatment capacity were used.

The ESFT only models the demand for face shields in critical medical and front-line applications, yet face shields are also increasingly seen as desirable in non-medical settings as a means of infection control among the general population [4]. Some airlines have mandated the use of face shields by passengers [5] and face shields are increasingly being adopted by businesses and institutions seeking to provide additional protection for workers who routinely come into contact with large numbers of potentially infected individuals. Demand for face shields (particularly of single-use designs) by the general population, could increase overall demand for face shields beyond levels predicted by the ESFT. This additional demand could also place stress on supply of face shields for critical medical and front-line applications [6]. At the time of writing, supplies of face shields are reportedly insufficient to meet demand in many locations [7, 8].

#### 1.1.2 Manufacturing and distribution challenges

Failures to provide sufficient face shields to those who need them have apparently been primarily due to limitations in manufacturing and distribution capacity. In the context of the COVID-19 pandemic, shortfalls in PPE provision are likely to be further exacerbated by disruptions to global manufacturing supply chains and distribution. These may be caused by transportation restrictions, social distancing measures, or worker illness, all of which can potentially lead to labour and material shortages and reduced manufacturing productivity, adversely impacting the timely production and distribution of essential PPE. Face shield shortfalls are likely to be especially high in less wealthy countries, which are often unable to compete with wealthier countries on a price basis to import PPE during a global shortage [8]. Scaling up domestic PPE manufacturing and distribution capacity in such countries, which are often less industrialised and more rural, may be prohibitively expensive and slow given current methods and technologies. The result is that many medical facilities in these areas are currently grossly under-equipped with respect to the PPE required to treat patients and protect healthcare workers in the context of the COVID-19 pandemic [8].

#### 1.1.3 Design adaptability

The provision of sufficient face shields is also hindered by the potentially diverse ergonomic, performance, and protection requirements of users. Some medical use-cases for face shields require that additional medical devices, such as surgical loupes, be worn underneath face shields, potentially requiring an adapted design suited specifically for this application. Furthermore, if the general population is increasingly required to wear face shields, designs which provide sufficient protection and comfortable fit for a variety of wearer headforms, including children, are required. Developing and testing novel face shield designs suited to different users and applications is technically challenging and time-consuming, potentially reducing the speed and volume at which face shields can be delivered to these populations.

In addition to variation in user requirements, differences in availability of raw materials, manufacturing equipment, and skilled labour in various regions may limit the ability of manufacturers to scale production of an existing face shield designs, a challenge already highlighted as impacting less wealthy countries especially severely. The growing Free and Open-Source Hardware (FOSH) movement may provide lessons in rapid, collaborative, distributed design, testing, and manufacturing of PPE in response to the COVID-19 pandemic in a diverse range of contexts [9].

#### 1.1.4 Environmental impacts

Many existing face shield designs are intended to be single-use, largely due to the challenges with ensuring efficient and effective decontamination. Given the enormous global demand for face shields projected over the course of the pandemic, high volumes of plastic waste, contained in clinical waste streams, are likely to enter landfills as a result. Assuming 50 grams of plastic are used for one single-use face shield, the consumption of 1.03 billion face shields over the coming 12 weeks could result in 52 kilotonnes of plastic waste entering landfill.

### 1.2 Opportunities in design innovation to address PPE shortages

New designs and manufacturing methods for face shields which are cost-effective, allow for rapid high-volume production, and are resilient with respect to supply chain disruptions could allow for increased production of face shields to meet predicted global demand. Challenges in local production of face shields in low-income regions could be addressed through the development of face shield designs which may be manufactured using a variety of available manufacturing methods, materials, and labour. Simple designs which may be replicated and adapted easily with minimal specialist knowledge could allow for manufacturers in both wealthy and low-income countries to modify the design to best suit the performance and ergonomic requirements of their intended user. Challenges in rapid global distribution, particularly to rural and remote areas, could be addressed through designs which may be transported in a space-efficient manner.

Designs which allow face shields to be safely reused could also significantly reduce global demand for face shields, reduce plastic waste, and result in significant cost savings. Assuming they could be safely reused over 10-50 shifts at equivalent cost and material consumption, reuseable face shields could reduce demand by 90-98%, resulting in global PPE cost savings of approximately $560-610 million, and the prevention of approximately 47-51 kilotonnes of plastic from entering landfill over the coming 12 week period. Reuse could also help to ensure sufficient supply of face shields during interruptions to manufacturing and distribution which, if only single-use face shields were available, would result in shortages or require significantly higher stockpiling of face shields to prepare for. Challenges in reusability of face shields could be addressed through designs which minimise the use of materials which are difficult to decontaminate, are designed with geometries that result in minimal trapping of soiling and maximal access by decontaminating agents, and are easy to visually inspect for soiling.

**Fig 1:**
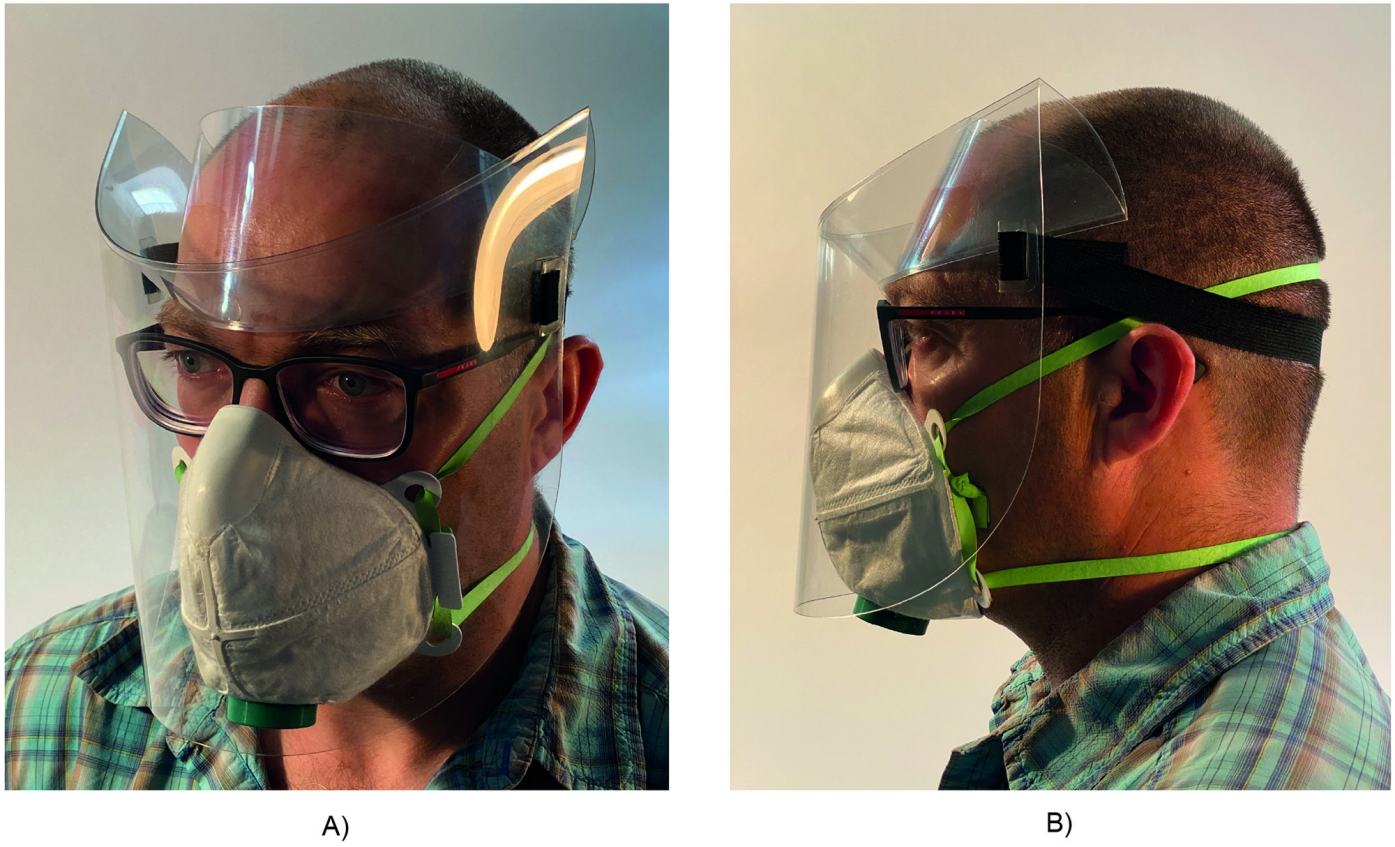
Curved crease origami face shield design. (A) three-quarters view (B) profile view. The individual in this figure has given written informed consent (as outlined in the PLOS consent form) to publish this image.

This paper presents a design (Fig 1) for a face shield for infection control which consists of a single folded sheet of flexible, clear, fluid-impermeable plastic, strap holders made using the same plastic, and an elastic strap. Subject to the validation of an approved decontamination procedure, this design is also likely to be reusable. A range of feasible manufacturing methods are presented, demonstrating the ability for the design to be produced using a variety of available machinery and labour. A flexible physical-digital iterative design methodology is presented which allows designers to adapt it to specific ergonomic, protection, and other requirements as appropriate for their local context and application.

## 2 Design requirements of face shields for infection control

### 2.1 Performance requirements of face shields for infection control

The function of face shields for infection control is to protect the wearer from liquid droplets and sprays which may contain infectious agents. Face shields must also allow other PPE, including respiratory protection (such as respirators or surgical masks) and additional eye protection (such as goggles) to be worn in conjunction with the face shield. Specific design and performance requirements for face shields for infection control vary by jurisdiction.

The performance requirements for face shields for a variety of applications are described in European standard EN 166. The face shield design presented in this paper was tested to the British Standards Institute’s PPE Technical Specification 2020/403 for Healthcare Professionals during the COVID-19 Pandemic, which cites performance requirements outlined in EN 166, listed below:

- BS EN 166:2002: 6.1. General Construction
- BS EN 166:2002: 6.2. Materials
- BS EN 166:2002: 6.3. Headbands
- BS EN 166:2002: 7.1.1. Field Of Vision
- BS EN 166:2002: 7.1.2.2. Spherical / Astigmatic / Prismatic / Refractive Powers
- BS EN 166:2002: 7.1.3. Quality of Material And Surface
- BS EN 166:2002: 7.2.4. Protection Against Droplets and Splashed of Liquids
- BS EN 166:2002: 7.2.8. Lateral Protection

The key requirement of face shields as described in EN 166:2002: 7.2.4. Protection Against Droplets and Splashes of Liquids, is the protection of the mucous membranes of the eyes from direct contact with liquids and sprays containing infectious agents.

### 2.2 Design requirements for face shields in the context of severe shortages

Due to the severe shortages of PPE experienced during the COVID-19 pandemic, revised recommendations have been made by a number of public health bodies concerning the use of PPE. The WHO, in particular, recommends the following “last resort temporary measures”, which may be “considered independently or in combination, depending on the local situation” when severe PPE shortages are likely to be experienced [6]:

1. PPE extended use (using for longer periods of time than normal according to standards);
2. Reprocessing followed by reuse (after cleaning or decontamination/sterilization) of either reusable or disposable PPE;
3. Considering alternative items compared with the standards recommended by WHO.

The above recommendations have a number of implications for the purpose of the development of new face shield designs which may be used in contexts experiencing severe PPE shortages. The recommendation that PPE be worn for longer periods increases the importance a comfortable fit for as wide a variety of wearer physiologies as possible, and the minimisation of excessive pressure, chafing, scratching or other sources of discomfort or potential injury which may result from wearing face shields for extended periods. The recommendation that reprocessing of face shields be considered necessitates face shield designs which may be effectively decontaminated, ideally in a labour and resource-efficient manner.

## 3 Precedent face shield designs

Since the beginning of the COVID-19 pandemic, a variety of organisations and institutions have developed novel face shield designs and production methods. Based on a survey of these designs, four broad categories of face shields were identified. These categories are distinguished largely by the design, manufacturing procedure, and materials used in the face shield suspension system, which supports the transparent visor that provides the primary protective function. It was observed that while suspension systems varied widely, visor components were largely similar between designs, typically consisting of an elastically bent sheet of flexible clear plastic.

**Fig 2:**
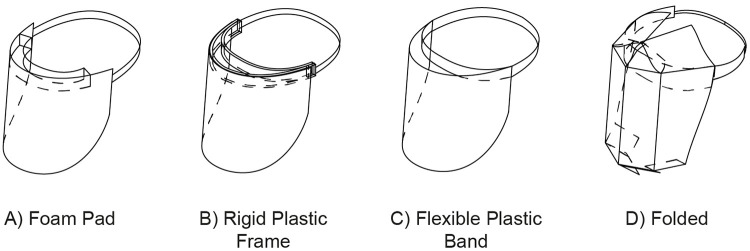
Face shield suspension system types. Simplified illustrations based on designs published by (A) [10] (B) [11] (C) [12] (D) [13].

The categories of face shields identified were the following: face shields which use a 1) foam pad, 2) rigid plastic frame, 3) flexible plastic band, or 4) contiguous folding of the visor sheet for their suspension systems, as shown in Fig 2. Table 1 presents a comparative analysis of the characteristics of the face shield designs surveyed. The features evaluated include fabrication methods, materials required, reusability, protection from above (top coverage), and the open-source provision of the digital data required for their fabrication.

**Table 1:** Characteristics of selected face shield designs grouped by suspension system.

	Rigid Plastic						Flexible Plastic					Folded		Foam Pad		
	Amazon [14]	UoN [15]	UoE [16]	UoS [17]	IESchool [18]	Prusa [11]	Foster+Partners [19]	Apple [20]	AmbroPlastic [12]	MTC [21]	Kitronik [22]	MIT [13]	ICD [23]	Nike [24]	RISD [25]	Addenbrooke’s [10]
**Fabrication method**																
3D printing	•	•	•	•	•	•										
Laser cutting							•	•		•	•				•	•
Die cutting									•			•				
Manual fabrication													•			
**Materials required**																
PLA	•	•	•	•	•	•										
PET / PETG	•	•	•	•	•	•	•	•		•		•	•		•	•
Acetate/PVC											•			•		
Elastic/rubber band	•			•	•	•						•	•			•
PP (strap/headband)		•					•		•	•	•					
Silicone rubber							•	•								
Foam							•							•	•	•
Staples													•			•
Adhesives														•		•
Adjustable string														•		
**Reusability**	•	•	•	•	•	•	•	•					•			
**Top coverage**		•			•		•			•		•	•	•	•	•
**Open source**	•	•			•			•			•		•		•	

### 3.1 Suspension systems

#### 3.1.1 Foam pad

The first category of face shields surveyed are those incorporating foam strips and an elastic headband as a means of supporting the visor in front of the face in a curved configuration. Manufacturing procedures described for these designs typically make use of manual assembly processes, resulting in reported production capacities of 1500 face shields per day at one institution [26].

While the use of a foam pad likely helps to provide a comfortable fit for a variety of wearer headforms, it has a number of disadvantages with regards to the face shield provision in the context of the COVID-19 pandemic. First, effective decontamination and visual inspection of soiling is likely to be more challenging in face designs incorporating foam pads. Porous open-cell foam material may trap infectious agents both inside the material, and at the interfaces of the material and the visor. Both of these locations are difficult to visually inspect. A preliminary study of decontamination of face shields incorporating foam in the headband region found that biological indicators placed in the foam were not successfully sterilised [27]. Decontaminating agents, including alcohol or chlorine-containing solutions may also degrade foams and the adhesives used in some cases to affix them to the visor [28]. These factors mean that face shields incorporating foam pads are unlikely to be safely reusable.

Furthermore, the relatively high number of unique materials required in some surveyed designs using a foam pad (plastic sheet material, adhesive-backed foam or foam and additional adhesive, fixings, or elastic strap) increases the sensitivity of production of such designs to supply chain disruptions caused by the pandemic. Finally, the inclusion of foam pads limits the distribution efficiency of face shields by increasing both their volume and potentially the volume wasted in packing for storage and transport. The worst-case packing thickness of such designs, equal to the combined thickness of the foam pad and the visor sheet material, is likely to be approximately 20-25 mm, which is significantly higher than other designs surveyed.

#### 3.1.2 Rigid plastic frames

A large number of the surveyed face shield designs used rigid plastic elements as a frame for supporting the visor and enforcing the desired surface curvature. In some instances, these plastic elements were manufactured using 3D printing technology. While 3D printing allows for straightforward reproduction of designs with low barriers to entry in manufacturing procedure set-up, it suffers from very slow manufacturing times. Reported fabrication rates of designs produced using 3D printing methods reached 1,000 face shields per week (approximately 150 per day), depending on the scale of the production facility [17]. Furthermore, 3D printed plastic elements are porous, potentially trapping infectious agents and making them difficult to disinfect [29]. A number of designs surveyed in this category also did not provide protection from above. 3D printing of top visors requires significant consumption of material, and could further slow production rates. Finally, depending on their geometry and rigid nature, rigid plastic frames, similarly to foam pads, may also limit packing efficiency for storage and transport. The worst-case packing thickness of such designs may range between 5-20 mm.

#### 3.1.3 Flexible plastic band

A third category of face shields are designs where some or all of the headband component is cut from the same transparent plastic sheet material as used for the visor. This allows for high-volume and straightforward manufacture using laser-cutting or die-cutting methods. Die-cutting manufacture allows for the highest production rate of face shields reported, at up to 90,000 shields per day at one facility [12]. Smaller-scale labs producing such designs using laser cutting have reported production rates of 1,500—3,000 shields per day [25]. The smaller number of unique materials in these designs also results in reduced sensitivity to supply chain disruptions. Designs using flexible plastic bands may benefit from significantly higher packing efficiency than foam pads and rigid plastic frames, because they may be flat-packed for storage and transport and assembled into their three-dimensional configurations when being opened for use. This yields worst-case packing thicknesses approaching the thickness of the sheet material, or 0.5-1.0 mm. A limitation of the designs surveyed in this category is their lack of protection from liquid splashes and sprays from above.

#### 3.1.4 Contiguous folding

Finally, a small number of designs [13, 23, 30, 31] have explored the use of folding techniques to provide protection from liquids and sprays from above the face, by using a single contiguous sheet of transparent sheet material as both a front and top visor, and as part of the suspension system. These designs also benefit from high-volume manufacturing using laser-cutting and die-cutting. Folded designs also benefit from reduced supply chain sensitivity as they consist of a small number of unique materials. If transported in their flat-packed configuration, such designs also have worst-case packing thicknesses approaching the thickness of the sheet material, or 0.5-1.0 mm.

The designs presented in [13, 30, 31] use complex straight-line folding patterns which may only be practical to produce using mechanised methods of cutting and creasing. This potentially limits their adoption in areas with limited access to these mechanised production methods. [30, 31] further require the use of both plastic and paper in the shield, apparently bonded together using adhesives. [23], by contrast, uses curved folding of a single contiguous sheet to achieve a face shield which may be transformed from a flat sheet to a three dimensional geometry which conforms to the wearer’s head. The simplicity of the folding pattern and cutting boundary used in such a curved crease design make it producible using manual methods. However, [23] uses staples for headband strap attachment and to stabilise the geometry of the shield, posing a potential scratching hazard, and preventing headband strap length adjustment to suit different wearer headforms. By permanently affixing the panels of the shield to each other, these staples also prevent the design from being returned to its flat configuration after folding. Finally, permanent metallic fasteners such as staples likely limit reusability, because these could trap infectious agents, limit access of sterilising agents, and could corrode upon repeated contact with water when this is used for cleaning.

The design presented in this paper (Fig 1), shared publicly at around the same date as the similar design presented in [23], also uses a simple curved folding pattern to achieve a shield conforming to the wearer’s head, which may be manufactured using manual methods. This design is described in greater detail in Section 5. A potential challenge with folded face shield designs is developing folding patterns for a variety of wearer headforms and user performance requirements. In addition to reporting on the design developed as part of this work, this paper also presents an iterative physical-digital prototyping method for rapidly developing curved folding patterns of face shields for a variety of wearer headforms.

### 3.2 Observations on the state of the art of face shields for infection control

The above survey of existing face shield designs highlighted large differences in the production rates of face shields resulting from the use of certain manufacturing processes, ranging over nearly three orders of magnitude. The survey also highlighted the importance of material selection to enable effective decontamination of face shields for reuse. The inclusion of open-cell foams, 3D printed materials, and adhesives was identified as potentially preventing effective decontamination.

To illustrate the importance of high-volume production and the ability to effectively reuse face shields, it is worth considering the rate of production required to meet global predicted demand for face shields in the single-use and reuse cases. Assuming a production rate of 90,000 face shields per day from a known die-cutting facility [12] (the fastest rate of production of face shields known to the authors), approximately 137 such facilities globally would need to be operating at full capacity continuously to produce the required average number of face shields daily over the coming 12 week period. Assuming reuse over 10 or 50 times, only 14 or 3 such facilities, respectively, would be required to meet global demand, assuming optimal distribution.

The packing efficiency of face shields for storage and transport was also identified as an important consideration in design, with estimated packing efficiencies for the designs surveyed ranging over an order of magnitude. Individual packaging of face shields is also likely desirable for infection control. Packing film thickness for flexible packaging is likely to range from approximately 0.013 mm to approximately 0.076 mm [32]. Incorporating two layers of packing film (above and below) the shield in estimates of the worst-case packing thickness for face shields slightly reduces the estimate of the difference in packing efficiency between thicker and thinner face shield designs. It should noted here as well that reuse could significantly reduce packaging waste, and environmental impacts due to transportation of face shields.

Many shield designs surveyed do not provide protection from liquids and sprays from above, potentially limiting their fitness for purpose. Finally, the use of a small number of unique materials in designs was identified as a means for limiting the sensitivity of face shield manufacturing to supply chain disruptions.

## 4 Opportunities in sheet-material manufacturing for PPE production

### 4.1 Advantages of sheet material fabrication

The manufacturing and distribution of products from sheet materials has a number of advantages relevant to the production of face shields during the COVID-19 pandemic. Raw sheet materials can be rapidly produced at high volume and transported efficiently to fabrication sites due to their high packing efficiency in rolls or stacks. As identified in the previous section, products made from sheet materials may also in some cases be transported in their flat configurations with high packing efficiency. Numerous manufacturing methods exist for cutting and creasing of thin sheet materials, ranging from completely manual methods (hand-cutting, creasing, and folding) through to computer-controlled cutting methods (laser, waterjet, and drag knife cutters) and a variety of pressing methods (die-cutting and stamping). This flexibility with respect to manufacturing methods is a key advantage in the context of the provision of PPE during the COVID-19 pandemic, where distributed manufacturing approaches making use of locally available machinery and labour could help to meet PPE demand in developing and remote regions.

When combined with folding, in an “origami” approach, sheet material manufacturing techniques may be used to produce complex, varied, and high-performance mechanisms and structures. Diverse available folding patterns can achieve a variety of functionalities, such as deployability, load-carrying capacity, and kinetic energy dissipation behaviour. Origami folding techniques have been increasingly studied and adopted across a wide range of disciplines, including medicine, aerospace, and architecture.

### 4.2 Curved-crease origami

Curved-crease origami are a class of origami structures that possess curved fold lines, rather than the straight fold lines seen in typical origami. Curved-crease origami allow transformation of flat sheet materials into complex three-dimensional forms which possess curved surface regions, as their unique curved folds cause bending in the sheet material during the folding process [33, 34].

**Fig 3:**
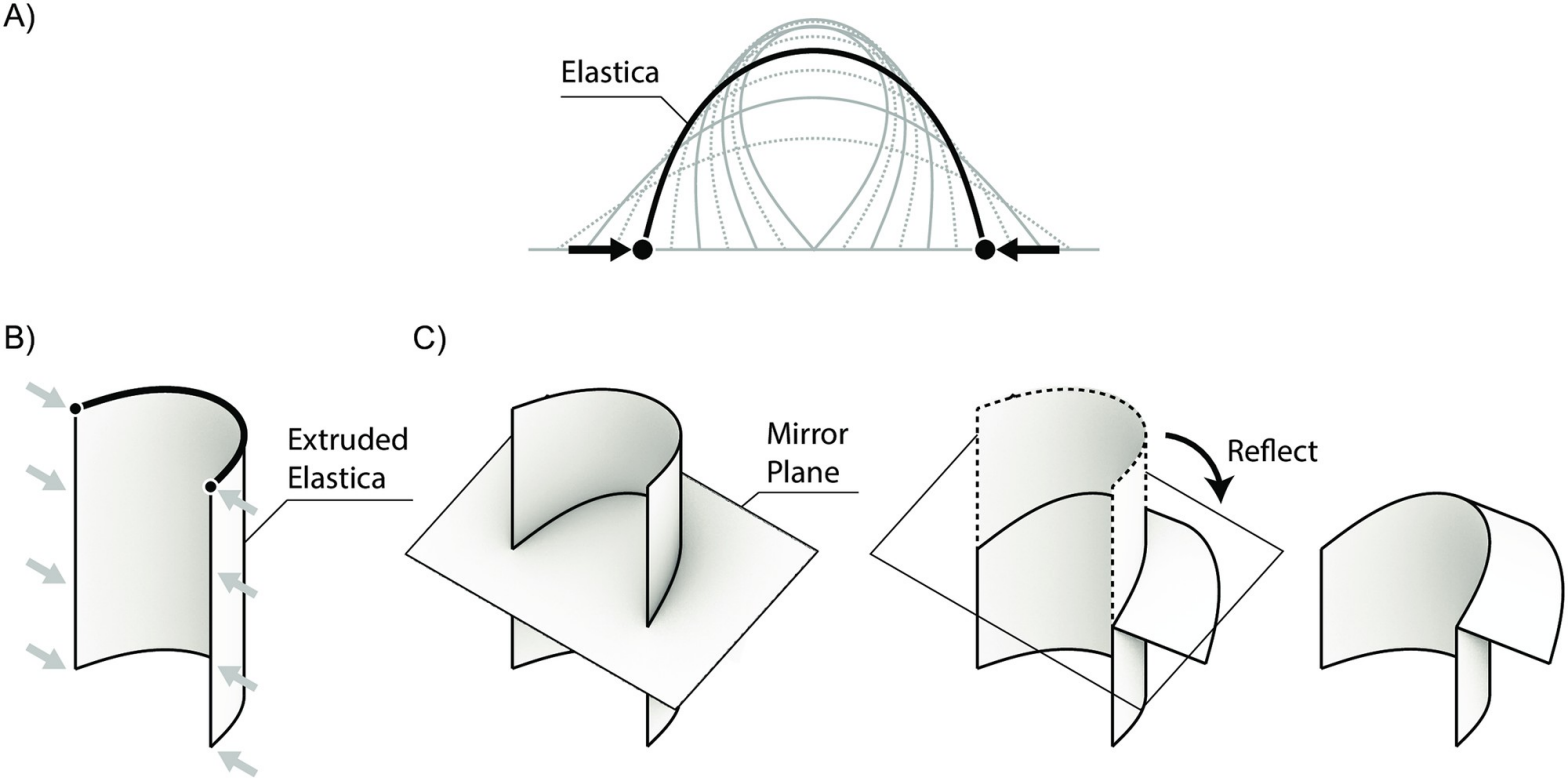
Elastica curved-crease origami surface generation approach. A) Elastica B) Extruded elastica C) Curved-crease origami generation.

Recent research has demonstrated concise analytical models for designing the three-dimensional surface geometries of certain special cases of curved-crease origami [35]. These cases assume an “elastica” surface curvature corresponding to a minimum energy configuration of an elastically-bent slender rod, Fig 3A [36]. This assumed curvature can be extruded and reflected to generate a developable three-dimensional surface [37], Fig 3B, with the surface then unrolled to give the curved fold lines required for manufacture from an elastic sheet material. Parametric control over the elastica curve and the position and orientation of mirror planes allows for precise control over the size and shape of the generated three-dimensional form, or over the size of the two-dimensional sheet material required for production.

## 5 Curved-crease origami face shields

**Fig 4:**
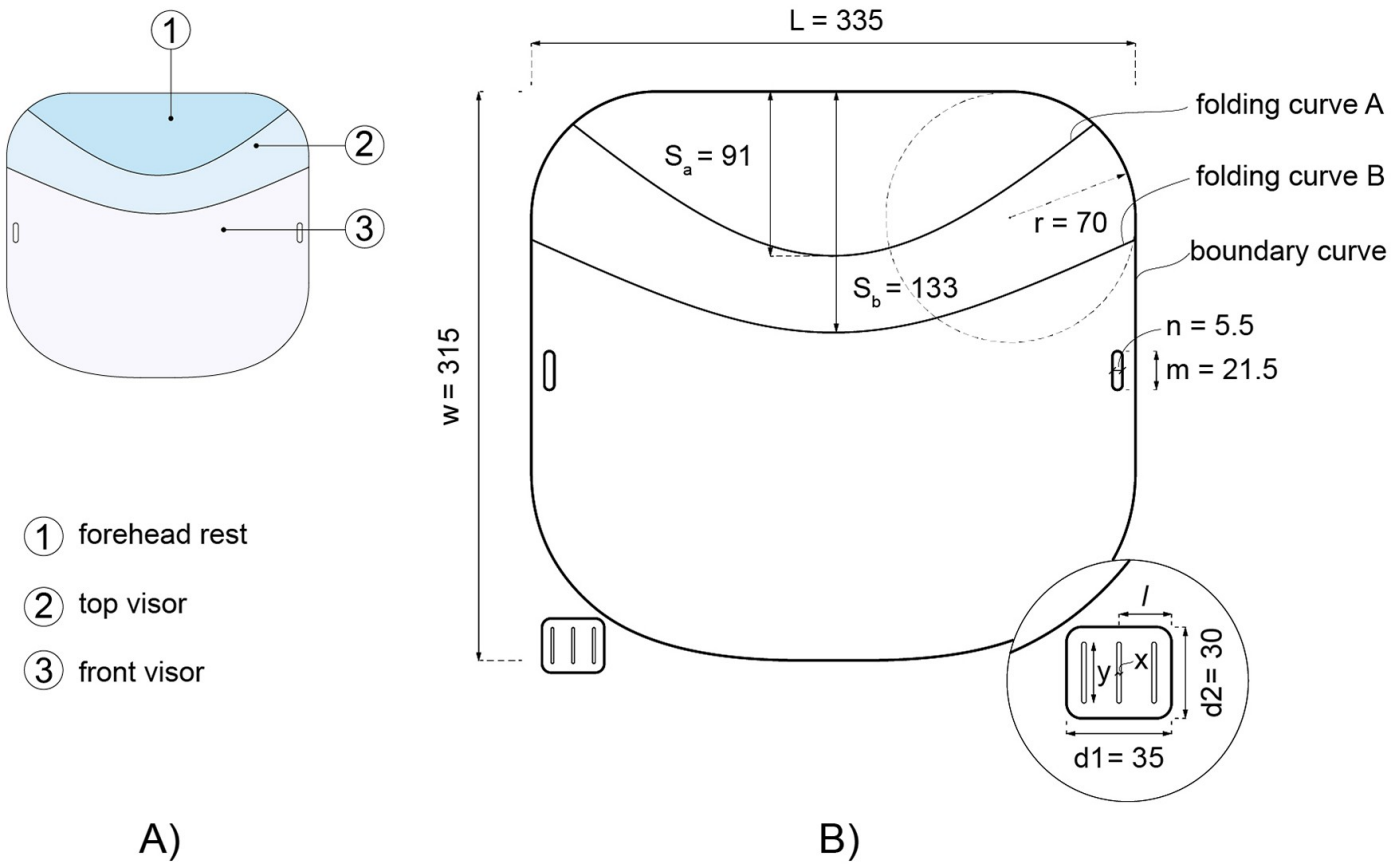
(A) Curved crease origami face shield comprised of three surfaces: 1) a forehead rest; 2) a top visor; and 3) a front visor. (B) The design parameters of the unrolled geometry of the face shield (dimensions in mm).

The face shield design presented in this paper (Fig 4) uses an elastica surface curved-crease origami approach involving the creation of two curved folds in a sheet of flexible transparent plastic. When folded along the two curves, the sheet is transformed into three contiguous surfaces (Fig 4A), which, together, perform the suspension and protection functions required of face shields for infection control. These three surfaces are 1) the forehead rest, which provides support for the face shield geometry on the forehead of the wearer; 2) a top visor which rigidly positions the shield with respect to the head attachment and protects the wearer from splashes, sprays, and aerosols from above; and 3) the front visor which protects the wearer from splashes, sprays, and aerosols from the front and sides. The simple curved folding pattern of this design (similarly to [23]) make it practical to produce using universally accessible manual methods, in contrast with the complex straight-line folding patterns required to produce the shields described in [13, 30, 31].

Rather than a permanently affixed headband strap as in [23], the design incorporates a removeable headband consisting of an elastic strap and strap holders, manufactured from the same plastic material used in the shield. This allows for strap adjustment to suit a variety of headforms, and permits the headband strap to be removed as part of potential decontamination procedures. The headband strap is attached to the front visor, as opposed to the interface of the top visor and forehead rest as in [23]. This stabilises the shield geometry when worn without the requirement for permanent fasteners. This property allows the design to be reversibly folded and unfolded from its two-dimensional flat-packed to three-dimensional worn configuration. The shield may be transported in its flat-packed configuration at high packing efficiency, and folded into its three-dimensional configuration by the user upon unpacking at its destination. After use, the shield may be subsequently flattened again for possible cleaning, storage, or transport. For infection control, these shields could be transported individually in flexible plastic film sleeves.

**Fig 5:**
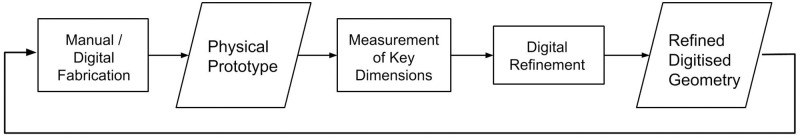
Iterative physical-digital design workflow used to develop curved crease origami face shield design.

Subject to validation of an effective decontamination procedure, the shield design could potentially be decontaminated for reuse by removing the headband, flattening the shield, and decontaminating the shield surface using a variety of cleaning and sterilising methods. The shield, apart from the removable, disposable headband, contains no materials or components (such as foam, 3D-printed elements, or staples) or geometric features likely able to trap infectious agents or limit access of sterilising agents. Finally, the design requires only two materials (PET sheet and elastic strapping), resulting in minimal supply chain sensitivity. This design was developed using an iterative two-stage procedure involving both physical prototyping and digital refinement and visualisation (Fig 5), described in the following sections.

### 5.1 Physical prototyping

Physical prototyping of the face shield design presented in this paper used manual cutting, creasing, and folding methods to rapidly produce prototypes for evaluation. This procedure was used to determine the approximate geometry of the boundary curve, folding curves, and the location and dimensions of the strap attachment holes of the face shield. This process, through which novel face shield geometries could be manufactured and evaluated within five to ten minutes, allowed for rapid evolution of the design to meet key performance requirements. Physical prototyping was conducted first using cardboard, later using 440 micron acetate plastic sheeting, and finally using 510 micron polyethylene terephthalate (PET) sheets.

#### 5.1.1 Curved foldlines

Physical prototyping was first used to determine the approximate location of the two curved foldlines in the face shield design. The position and shape of the foldlines affect the fit, protection, and mechanical behaviour of the face shield. A key consideration of the design is the angle between the forehead rest portion and front visor portion of the shield, which arises from the relative orientation of the two foldline reflection planes in an assumed elastica surface curved crease origami rationalisation of the face shield design. Experimentation through physical prototyping determined that in order to allow the forehead rest to adapt comfortably to the sloping profile of the human forehead, while orienting the front visor such that it did not project too far out from the face, a deviation angle of approximately 30 degrees between the forehead rest and front visor was effective.

A second key consideration for the design is the position of the front visor with respect to the face. The front visor must be far enough from the face to allow primary PPE (goggles and respirator or surgical mask) to be worn comfortably underneath the face shield, but not so far away from the face that protection from splashes and droplets is compromised. During physical prototyping it was also observed that the distance of the face shield from the face also influenced the likelihood of fogging, caused when water vapor suspended in exhaled gases condenses onto the inside of the front visor. Fogging increased when the front visor was closer to the face likely due to the reduced ability for vapor to leave the volume between the wearers face and the front visor, prior to condensation occurring. The distance of the front visor from the wearer’s face is controlled largely by the distance between the two folding curves.

#### 5.1.2 Boundary curves

The design of the boundary cutting curves for the face shield was informed by protection and performance requirements, including that face shields: provide adequate lateral protection from droplets and splashes; not restrict movement of the head with respect to contact with the shoulders and chest; and have no sharp projections which could pose a scratching or cutting hazard, particularly during donning and doffing. Physical prototyping and preliminary testing of shield samples to EN 166 requirements informed the width and height of the sheet used for the face shield. To reduce the sharpness of the shield geometry at locations which could pose a scratching or cutting risk, the upper corners of the shield were filleted along a circular profile, a geometry chosen for ease of replicability using non-digital fabrication methods. The bottom corners of the shield design follow a freeform curve chosen to balance protection and freedom of movement requirements. The boundary geometry also influenced fogging behaviour, with larger shields, particularly those with significant extension of the front visor below the chin, resulting in greater fogging.

#### 5.1.3 Strap hole attachment location

The location and dimensions of the strap attachment holes was found to significantly influence the geometry of the face shield when worn. Physical prototyping determined that higher strap attachment hole locations caused the front visor surface to spread laterally, assuming a more flat curvature towards the bottom of the shield. Lower strap locations resulted in the face shield geometry assuming a tighter surface curvature, particularly towards the bottom of the shield.

### 5.2 Digital design refinement

The aim of the physical prototyping procedure described in the above sections was to produce an approximation of the final geometry of the boundary curve, folding curves, and strap attachment hole locations and dimensions which were likely to result in designs satisfying all performance requirements. The folding curves produced using manual fabrication methods were intended to correspond approximately to those created using an elastica curved-crease origami surface generation approach. This section describes a procedure whereby a digital parametric model incorporating elastica surface generation was used to generate refined digitised folding curves based on those generated using manual methods.

#### 5.2.1 Parametric geometry modelling

**Fig 6:**
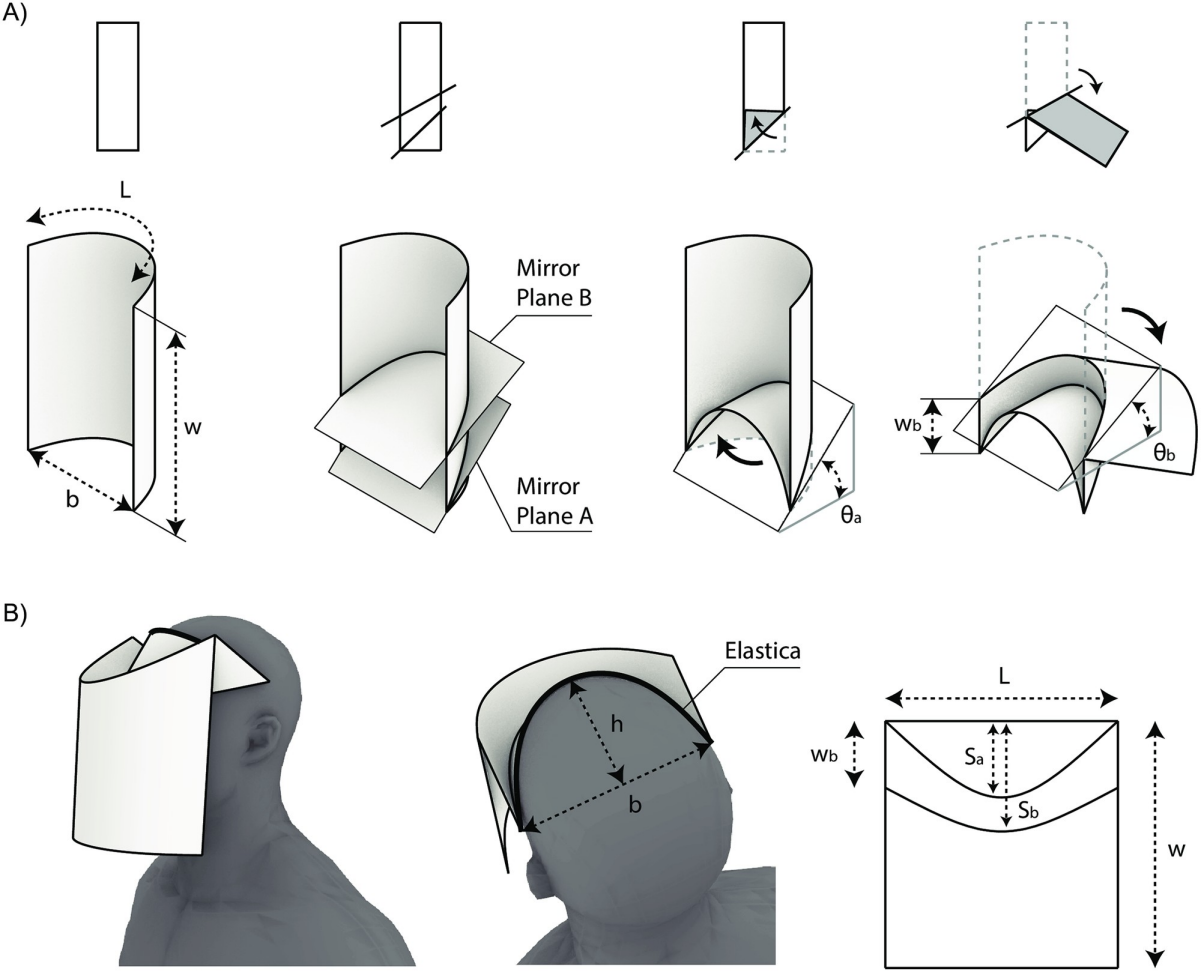
Analytical parametric model (A) construction sequence and key parameters. (B) Final folded form and pattern parameters.

The face shield was modelled using a three-dimensional parametric analytical model of a twice-mirrored extruded elastica shell (Fig 6). This parametric model provides a three-dimensional surface geometry prediction for the face shield design from six parameters, as shown in Fig A. These include:

- *L*: Horizontal sheet length.
- *w*: Vertical sheet length.
- *b*: Distance between the side edges of the face shield in its worn configuration, equal to the width of the wearer’s head at this point plus a gap.
- *θ*_*a*_: Inclination angle of Mirror Plane A, where Mirror Plane A intersects with the top corners of the sheet.
- *w*_*b*_: Distance of Mirror Plane B from the top of the sheet.
- *θ*_*b*_: Inclination angle of Mirror Plane B.

The parameters *L* and *b* describe a unique elastica, corresponding to the arc length and distance, respectively, between the elastica’s end supports. In this study, elastica curves were generated in a Rhino-Grasshopper parametric CAD environment using an open-source numerical solver [38]. When extruded by a distance *w* (the vertical size of the sheet), and mirrored at Mirror Planes A and B, this elastica provides a prediction of the three-dimensional surface geometry of the face shield. Boundary curves and strap attachment holes are assumed to be cut from this surface without altering its curvature. Importantly, this elastica surface generation approach assumes continuous translational restraint along the two edges in the extrusion axis (Fig 3B). This differs from the support conditions of the actual face shield in its worn configuration, which is supported by the wearer’s forehead against the forehead rest surface, and the elastic headband connected to the strap attachment locations. As will be shown in the following section, when the strap attachment holes are positioned through iterative physical prototyping such that the change in curvature of the front visor along its height is minimised, the above elastica surface generation approach is sufficiently accurate to reasonably predict the actual geometry of the face shield when worn.

To generate the refined three-dimensional geometry of the face shield, key design parameters were measured from the physical prototype and applied to the digital parametric model. Six length parameters may be directly measured from the physical prototype, including *L*, *w*, *b*, *w*_*b*_, and the distances *s*_*a*_ and *s*_*b*_, which are the distances from the top of the sheet to the mid-points of folding curves A and B respectively. The approach taken to generate the mirror plane angles *θ*_*a*_ and *θ*_*b*_ is to choose these values such that the mid-point rises of the digitally-generated folding curves are equal to the mid-point rises *s*_*a*_ and *s*_*b*_ of the folding curves measured on the physical prototype. Eqs 1 and 2 relate *θ*_*a*_ and *θ*_*b*_ to *s*_*b*_ and *s*_*b*_.

$$\begin{array}{c} \theta_{a}=\tan^{-1}\left(\frac{s_{a}}{h}\right) \end{array}$$ (1)

$$\begin{array}{c} \theta_{b}=\tan^{-1}\left(\frac{s_{b}-w_{b}}{h}\right) \end{array}$$ (2)

where *h* is the height of the unique elastica determined by *b* and *L* [39]).

As implemented in the Rhino-Grasshopper environment, this parametric geometry modelling tool is able to generate 3D geometry visualisations with sub-second response times on a typical personal computer, allowing for effectively real-time exploration of design options. The geometry generated using this digital refinement procedure was subsequently used to manufacture the face shield using digitally-enabled fabrication methods. Multiple iterations of manual physical prototyping, digital refinement, and digital fabrication were used to refine and generate the final design presented in this paper.

#### 5.2.2 Geometry validation

**Fig 7:**
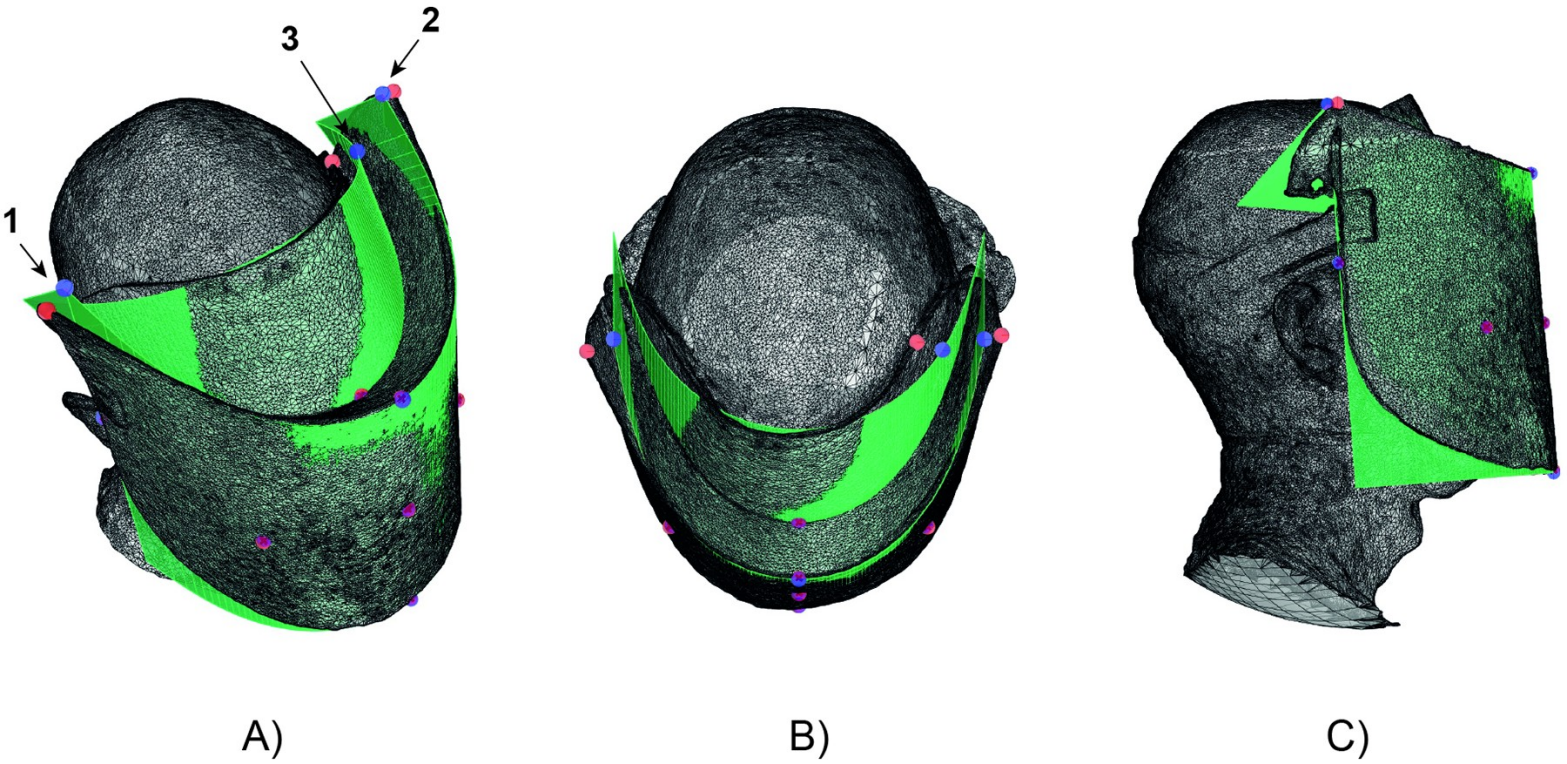
Comparison of predicted geometry with 3D photogrammetry scan of face shield on wearer’s head. 1) top right corner extraction point 2) top left corner extraction point 3) manually selected extraction point at top edge of forehead rest.

To evaluate the usefulness of the analytical mirrored elastica curved-crease origami approach for prediction of the geometry of the face shield, a 3D scan using photogrammetry was performed of a face shield on a wearer’s head (Fig 7). 120 photographs were taken of the face shield on the wearer’s head at approximately 0.5-1.0 metres distance. The photogrammetry software Metashape Pro was used to generate a 3D scan mesh representation from the images. The 3D scan mesh was scaled using a physical measurement of the widest point of the wearer’s ears in the horizontal axis orthogonal to the wearer’s viewing direction (195 mm) and the corresponding points on the 3D scan mesh. The 3D scan mesh was positioned and oriented such that the vertical centreline of the front visor of the 3D scan mesh and analytically predicted geometry coincided. The 3D scan mesh was rotated about this axis to an angle such that the perimeter of the visor in the 3D scan mesh roughly coincided with the corresponding locations on the analytically predicted geometry.

The deviation of the 3D scan mesh from the analytically predicted geometry was measured by comparing the scan mesh with the surface prediction at 11 extraction points on the generated surface, indicated as blue spheres in Fig 7. The extraction point at the top edge of the forehead rest (indicated as 3 in Fig 7) was chosen manually to determine the estimated maximum deviation of the forehead rest 3D scanned surface from the predicted geometry. For all of the extraction points except the top right and left corners (indicated as 1 and 2, respectively in Fig 7) and the top of the forehead rest, the corresponding closest points on the mesh were found automatically. To account for increased thickness of the 3D scan mesh due to photogrammetry artifacts at these locations, and the more extreme geometric deviations at the top corners and top of the forehead rest, corresponding points on the mesh at these three locations were chosen manually to correspond to the centre of the scan mesh thickness and the correct position on the 3D scan mesh geometry edge. All corresponding points on the 3D scan mesh are indicated as red spheres in Fig 7.

The distance between the between the 3D scan mesh and predicted geometry at all extraction points except for the top corners and top of the forehead rest was less than approximately 2.5 mm (approximately 5 times the thickness of the PET sheet). At the top right and left corners of the face shield, deviations between the 3D scan and the predicted geometry were greater, at approximately 15 and 11 mm, respectively. At the extreme point selected at the top of the forehead rest a deviation of approximately 14 mm was measured.

The above results demonstrate that the analytical prediction corresponds reasonably well to the true geometry of the face shield as measured using the 3D scanning method employed. The differences between the actual face shield and the analytical prediction are primarily due to the differences in support conditions in the analytical model compared to the actual face shield, as discussed in the previous section. The analytical shape prediction was also simplified to assume a rectangular boundary and no internal cuts, both of which would alter panel bending stiffness in the physical face shield.

The reasonably good correspondence between the analytical prediction of the geometry of the face shield and its true geometry as measured with a 3D scan suggest that the parametric modelling approach used to predict and visualise the geometry of the face shield is likely to be useful in the design process of future iterations and adaptions of this face shield. It is important to note here that several rounds of iterative prototyping may be required to determine strap attachment locations which result in minimal change in surface curvature of the front visor along its height, thus ensuring closest agreement with the analytically predicted geometry. A further factor which was found to potentially influence the geometry of the face shield when worn was the orientation of the sheet material in the face shield with respect to its rolling direction when packed for transport. The 510 micron PET sheet used in this study exhibited some residual curvature due to plastic deformation from being rolled. This could potentially influence the geometry of the face shield if the rolling direction during storage and transport coincides with the bending direction in the face shield’s worn configuration.

### 5.3 Rapid physical-digital prototyping approach for future face shield designs

The above approach using both physical prototyping and parametric digital design refinement may be used to rapidly explore and refine further iterations on the design presented in this paper. In particular, using the above approach, designers may straightforwardly adapt the design to different wearer head-forms, or make alterations to the geometry as needed to accommodate specific protection, ergonomic, comfort, or other requirements.

**Fig 8:**
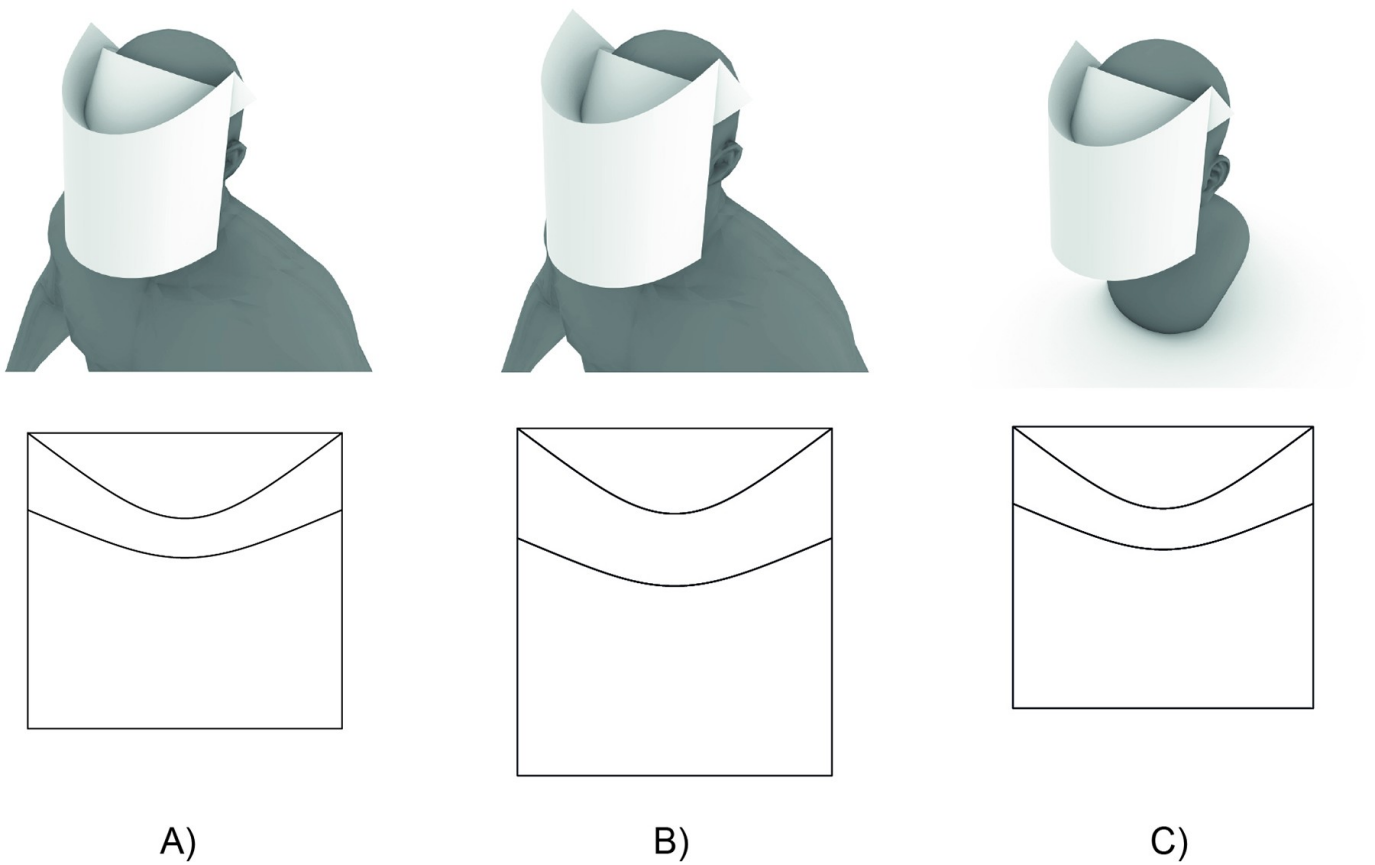
Variants of curved crease origami face shields based on the design presented: (A) original (B) variant with increased frontal clearance (for example to accommodate the wearing of surgical loupes underneath the face shield) (C) variant adapted for child (6-10 years).

Fig 8 illustrates two hypothetical adaptations of the design presented, using adjustments to the design parameters (shown in Table 2) to rapidly visualise the predicted geometries of the potential designs. The “Original” design shown in Fig 8 is the design tested to the EN 166 standard and found to be fit for purpose for the standard adult headform described therein. While this headform likely approximately describes the ergonomic requirements of a large portion of the general adult population, some populations may require alternative face shield geometries. Fig 8B shows a variant of the design which has been modified to have increased clearance in front of the wearer’s face, for example to allow for the user to wear surgical loupes under the face shield. This increased clearance is achieved by increasing the parameter *w*_*b*_ by 35 mm, to increase the extension of the top visor, thereby moving the front visor forward. As this also translates the front visor up, the vertical length of the shield *w* was increased by 55 mm to retain the same protective coverage around the chin. All other parameters remained unchanged. Fig 8C shows an adaptation of the design to a child (6-10 years) head form. In this variant, the width *L* of the sheet, and the target edge-to-edge width of the shield in worn configuration *b* were reduced to accommodate the smaller width and circumference of the child headform. The height *w* of the shield was also reduced to eliminate excessive extension of the bottom of the shield below the child’s chin. All other parameters remained unchanged. These alternative design variants have not been tested or certified, but are shown to demonstrate the adaptability and potential utility of the design process presented in this study. These designs are not intended to be representative of face shields required for all populations but rather illustrate potential modified designs suitable for specific populations potentially not well served by the “Original” face shield design presented in this paper.

**Table 2:** Parameters used for face shield design variants shown in Fig 8.

	mm	mm	mm	mm	Degrees	Degrees
	*L*	*w*	*b*	*w*_*b*_	*θ*_*A*_	*θ*_*B*_
Original (Fig 8A)	335	315	190	82.0	37.2	23.1
Increased Frontal Clearance (Fig 8B)	335	370	190	117	37.2	23.1
Child (6-10 yrs) (Fig 8C)	320	300	180	82.0	37.2	23.1

Generally, the key parameters which may be adjusted to adapt the geometry of the face shield to diverse wearer headforms are likely to be the horizontal sheet length *L*, which may be reduced to correspond to the reduced circumference of smaller wearer headforms, the vertical sheet length *w*, which controls the downward extension of the front visor, and the parameter *w*_*b*_, which controls the size of the top visor, which could be used to vary the distance of the front visor from the wearer’s face as needed. The parameter *b*, corresponding to width of the wearer’s head plus a gap, should also be adjusted accordingly for smaller or larger wearer headforms to enable accurate geometry prediction using the analytically generated face shield surface.

## 6 Manufacturing procedures for curved-crease origami face shields

A key objective of this work was the development of a design which could be produced using a variety of manufacturing methods, and a range of plastic sheet materials, to enable the rapid scaling of global production in the disrupted COVID-19 manufacturing and supply chain environment. A number of manufacturing procedures (summarised in Table 3) were explored as part of the design and prototyping process. The various manufacturing processes trialled used different techniques to produce cuts along the face shield boundary curves and at the attachment holes, as well as different methods for creating creases along the folding curves. For all methods trialled, folding of shields along creasing curves, folding and threading of straps, and attachment of straps was performed manually. Prototyping was explored in several thermoplastic sheet materials and thicknesses.

**Table 3:** Characteristics of manufacturing procedures for curved crease origami face shields, where accessibility and quality each may be “low”, “medium”, or “high”.

Method	*s / shield*	Accessibility	Quality
	Speed		
**Cutting**			
Manual	60-180	Universal	Low-Medium
Laser	45	Medium	Medium
Die	0.3*	Low	High
**Creasing**			
Manual	60-120	Universal	Low-Medium
Mech. Pressure	30-60	Medium	Medium
Partial-Depth Laser	*15-45*	Medium	Low
Complete-Depth Laser	*30-90*	Medium	Low
Die	0.3*	Low	High
**Folding**			
Manual	30-90	Universal	Low-High
**Strap Attachment**			
Manual	60-180	Universal	High

“Accessibility” refers to accessibility of manufacturing method globally to those seeking to manufacture face shields, and may also be denoted as “Universal” where methods can be reasonably expected to be ubiquitously accessible. “Quality” characteristics are discussed in the text. Italicised speeds refer to speed estimates for manufacturing procedures not tested in-depth as part of this work. *Die cutting and die creasing may be performed as part of a single procedure.

### 6.1 Material selection

Initial rapid prototyping of the face shield geometry was conducted using cardboard and cardstock sheets, and manual cutting and creasing methods. Once a rough geometry for the face shield design was identified, prototyping continued using thermoplastic sheets. Thermoplastic sheets are widely used as materials for face shield visors due to their optical properties, acceptable mechanical performance, light weight, cost and ease of disposal or re-use (as appropriate for the application). The specific selection of the type of thermoplastic for use in face shields is further based on properties such as mechanical behaviour, optical quality, chemical resistance, and impact and scratch resistance. A wide range of thermoplastics have been utilised in recent face shield designs, including polycarbonate [13], cellulose acetate [11], polyurethane [24], and PET [40], and PETG [13, 19]).

The design and fabrication methods presented in this paper were tested using transparent sheets of 440 micron cellulose acetate and 510 micron PET. Prototyping was also briefly conducted using 250 micron PET, however this material failed easily in a brittle manner near strap attachment holes, and was therefore not used further. Further work should explore the manufacture of these face shields using other thermoplastic sheet materials.

### 6.2 Cutting of boundary curves and strap holes

#### 6.2.1 Manual cutting

During initial physical prototyping, the cutting of boundary curves in plastic sheets was performed using scissors, and the cutting of strap attachment holes was performed using a utility knife. Strap attachment holes were also in some instances produced using manual hole punchers. To produce consistent and accurate cuts, tracing was used to transfer the geometry of the boundary curves and strap attachment holes to the plastic sheet. An individual shield could be cut manually in approximately 1-3 minutes. These methods, while slower and less accurate than mechanised methods, produced shields of acceptable quality for use. Specifically, manual cutting of the boundary curve was found to approximate the desired target geometry well enough so as to not affect coverage performance. However, care should be taken with manual cutting of the boundary curve so as not to introduce sharp projections (which could pose a scratching hazard) or slits (which could pose a hair trapping hazard. Furthermore, when manual cutting, care should also be taken not to over-size strap attachment holes such that strap attachment clips are able to detach from the front visor during use. Manual cutting methods may be particularly appropriate in settings where cutting machines may be unavailable or prohibitively expensive, but labour is available and relatively inexpensive.

#### 6.2.2 Mechanised cutting

A variety of mechanised cutting methods were explored for producing boundary curve and strap attachment hole cuts. A digitally-controlled laser cutter performed cuts rapidly, performing all necessary cuts for a given shield within approximately 45 seconds. Laser cutters are relatively widely available, making them a fairly accessible tool for local production of face shields. Shields cut using laser cutting were of good quality, passing required performance tests for face shields according to European standards mentioned in Section 2.1. However, laser-cutting produced char at the cut boundaries, potentially requiring some manual cleaning before packaging. Furthermore, we hypothesise that laser cutting may introduce local brittleness in PET sheet near cuts through local heating of material caused by the laser cutting process. Finally, laser cutting settings must be carefully calibrated, and sheets must be carefully positioned in order to ensure consistency of cuts, potentially compromising quality in the event these conditions are not successfully controlled.

Cutting of boundaries using a die in a manually operated bench-top hydraulic press was also explored. A key challenge observed with this approach was ensuring a complete cut through the sheet along the entire boundary curve. For the relatively small 10-tonne bench-top hydraulic press available, achieving a complete cut sometimes proved difficult due to the challenge of providing sufficient pressure to the cutting die at large distances from the hydraulic ram. For larger presses, and for high-volume die-cutting machinery, achieving complete cuts should pose no challenges, as has been demonstrated by existing similar face shield manufacturing projects [12, 13]. Assuming 90,000 shields produced using die-cutting at a known facility over an 8 hour work day, the speed of die-cutting per shield is estimated here as 0.3 seconds per shield. While fast, and likely consistently producing shields of high quality, die cutting machinery is less widely accessible than other manufacturing methods, limiting its use in some areas.

### 6.3 Creasing

The face shield design presented in this paper depends on the ability to consistently fold plastic sheets along predetermined curves. Controlled folding along curves may be aided through the creation of creases prior to folding. Informally, creasing is a process whereby a hinge may be produced in an otherwise stiff sheet material, allowing it to be folded at the crease. This hinge is produced by locally reducing the bending stiffness of the sheet material along a curve.

Creases in the presented face shield design must allow for multiple cycles of folding and unfolding, to enable the transformation of the face shield from its flat configuration to its three-dimensional worn configuration, and back to its flat configuration for cleaning, storage, and/or transport. To meet these design requirements, the number of folding and unfolding operations capable of being performed before a mechanical crease failure occurs should be maximised. A mechanical failure of the thermoplastic sheet is considered to render the shield unfit for purpose, as this break could compromise the ability of the shield to protect the wearer from infected liquids and aerosols, or could result in a scratching, cutting, or hair-trapping hazard. A variety of creasing methods for thermoplastic sheets for face shields were explored as part of this study.

#### 6.3.1 Manual pressure creasing

**Fig 9:**
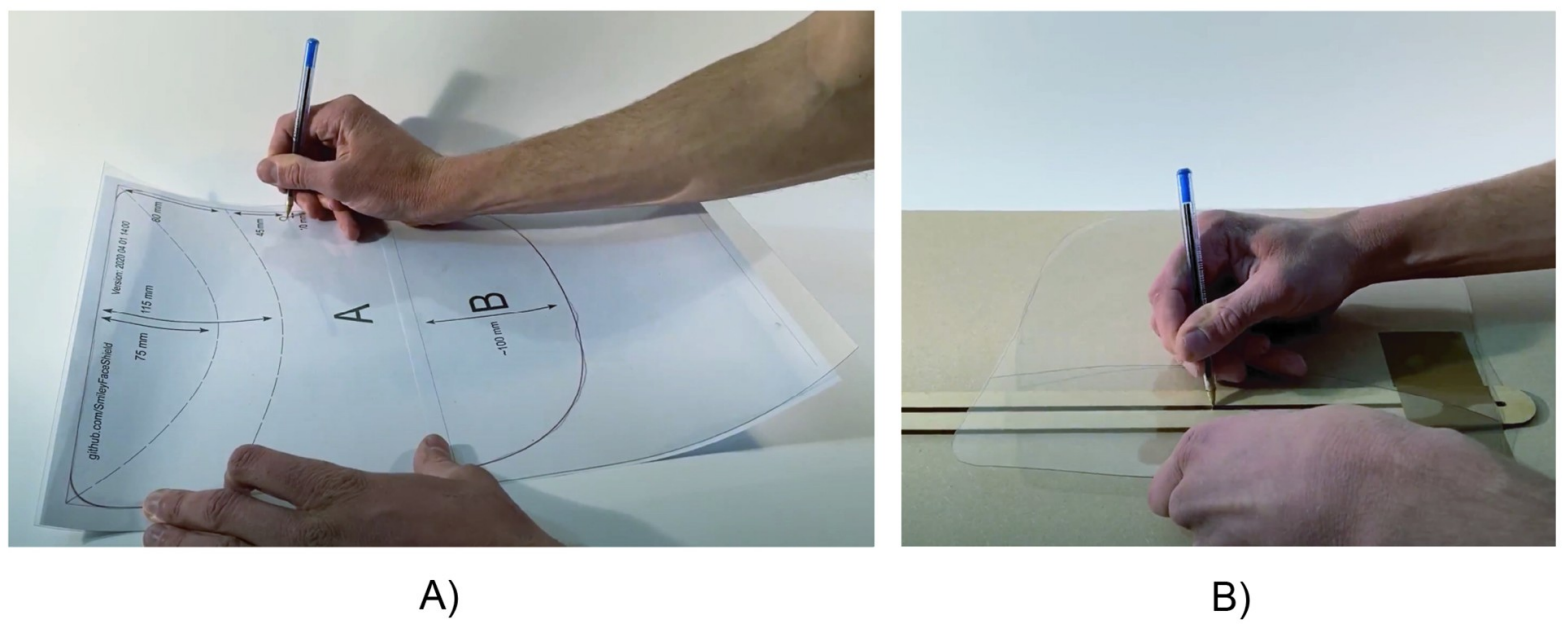
Manual fabrication procedures: (A) manual tracing of digitised boundary, folding, and strap attachment hole curves (B) manual creasing procedure using a ballpoint pen and channel.

The first method of creasing explored was the use of manual methods to induce local plastic deformation along the desired creasing curve by applying localised pressure to the sheet over a recessed channel using a hand-held tool (Fig 9). These manual creasing methods involved first tracing the creasing curves onto the sheet material for reference. Next, the sheet was positioned over a 1-4 mm-wide channel of greater than 2 mm depth, so that the sheet material did not reach the bottom of the channel when deformed under applied pressure. A blunt, soft tool was subsequently manually pressed into the sheet over the channel in a repetitive sliding motion, while the sheet was repositioned such that the resulting crease followed the target curves. The choice of creasing tool was found to significantly influence the mechanical performance of the resulting crease. Dull tools with relatively low material hardness were found to produce creases with better robustness than sharp tools made of harder materials.

The above manual creasing procedure could be completed in approximately 1-2 minutes per shield. This method was highly effective for rapid prototyping, and when conducted carefully using appropriate tools and recessed channels, could produce shields of acceptable quality. If this method were to be implemented at scale, however, dedicated quality control procedures might be desirable for ensuring the required mechanical performance of creases. In addition to ensuring that creasing implements are of appropriate dullness and softness, controlling the consistency of applied pressure is likely to be of key importance in such quality control procedures.

A key advantage of manual creasing is that when combined with manual cutting methods, this procedure can be performed with nearly universally available tools by users with little to no specialised skills. Because the fabrication procedure may be performed entirely on a flat sheet, a 2D cutting and creasing template (Fig 9A) may be easily used to reliably reproduce the shield geometry of designs which have been tested for their performance against product standards. In the course of this research, cutting templates for the face shield design developed in this work were distributed digitally and used to allow makers in other locations to produce the design with little to no specialist tools required. Distribution by non-digital channels, that is, postal mail, is also enabled by this approach, meaning that performance-evaluated shield design templates and the raw materials required to make them could be distributed to remote locations without internet infrastructure or 2D printing capability. This approach could be particularly valuable in less-developed regions, or in regions suffering from disruptions to communications infrastructure due to natural disasters or conflict. If 2D printing could be performed directly onto the plastic sheet material used for the shield, this could further simplify the manual cutting creasing process through the provision of visual cutting and creasing guides on the shield material itself.

#### 6.3.2 Mechanised pressure creasing

**Fig 10:**
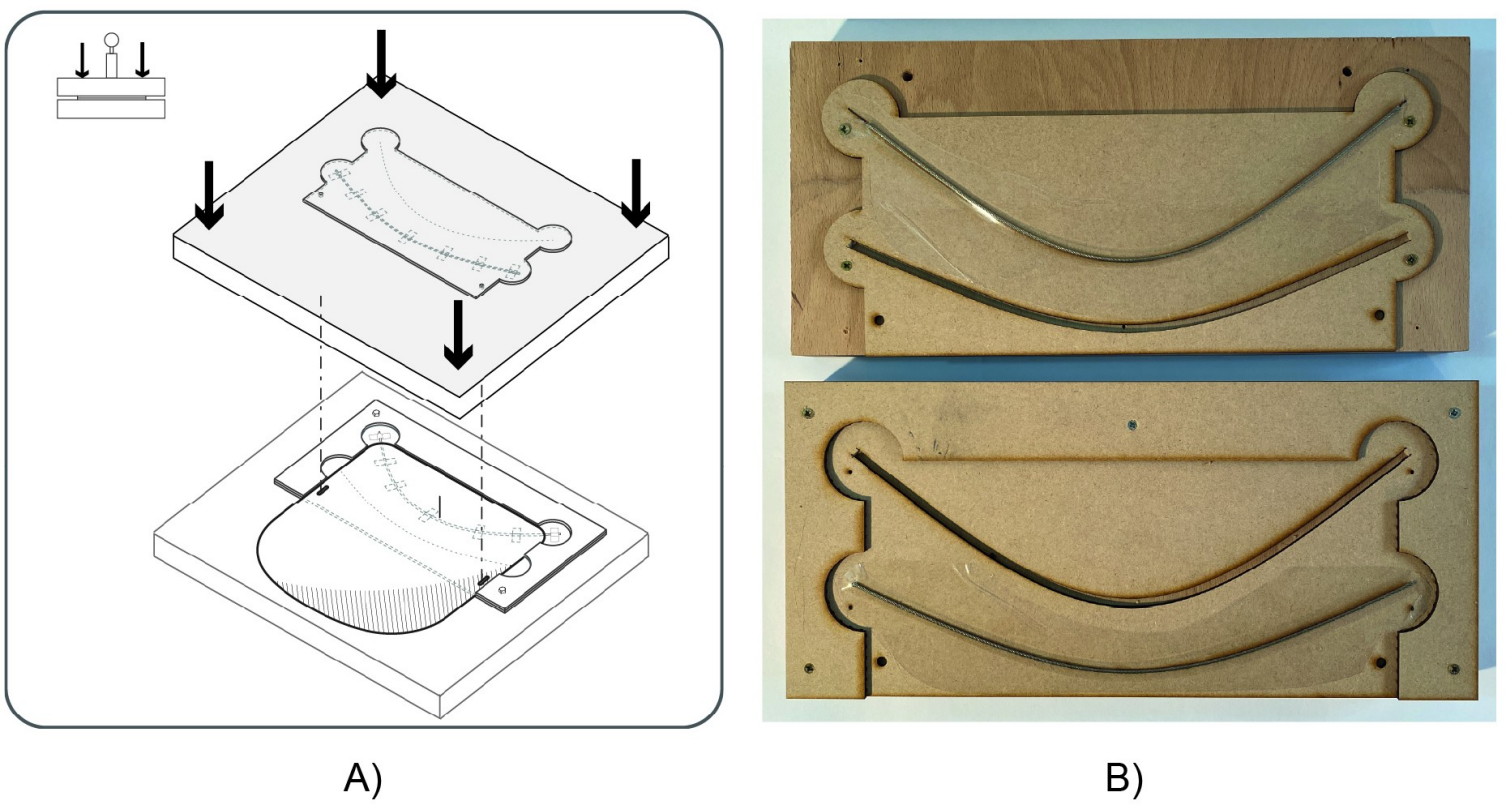
Die used for creasing with a bench-top hydraulic press (A) Illustration of pressing procedure (B) Photograph of prototype creasing die.

Another method explored to produce creases in thermoplastic sheets was the use of a creasing die on a bench-top hydraulic press. Dies (Fig 10) were produced by laser-cutting 4 mm medium-density fibreboard (MDF) sheets into a three-part template, and affixing a 2 mm wire rope to the top and bottom surfaces of the die opposite a 5 mm-wide receiving channel. Plastic sheets with boundary curve and strap attachment hole cuts already complete were placed between the top and bottom surfaces of the die, and the assembly was subjected to an overall applied force of approximately 60-70 kN. The wire rope and receiving channel caused a local deformation of the thermoplastic sheet resulting in a folding crease with a predetermined favoured folding direction. This method was found to produce consistent creases with excellent robustness. The procedure to insert, crease, and remove a shield using the above approach took approximately 30-60 seconds.

An alternative die was also tested using the same hydraulic press, using a curved creasing bar integrated into a cutting die. This method, while clearly promising in the context of high-volume die-cutting and creasing production, was not successful using the relatively low-capacity hydraulic press available for this study, because of the strong variation in applied pressure as a function of distance from the hydraulic ram. Specialised high-volume manufacturing machinery for combined cutting and creasing of PET sheets exists, suggesting that implementing a high-volume combined cutting and creasing manufacturing method for the design introduced in this paper would likely not pose significant technical challenges. The speed of die creasing is estimated here using the same approach as for die cutting.

#### 6.3.3 Partial depth cut creasing

Another creasing method explored as part of this work was the use of partial-depth cuts. Using lower power and higher speed settings on a laser cutter than would result in a complete depth cut, an “engraving” cut may be achieved which does not completely penetrate the material. The resulting reduction in cross-section results in a desired local reduction in bending stiffness at the crease, producing a hinge when folded.

An advantage of the above approach, whereby an incomplete depth cut is made by laser engraving, is that this procedure may be performed quickly (likely between 15-45 seconds per shield), and could be completed in conjunction with complete depth cuts required for boundary curves and strap attachment holes. A key disadvantage of this approach, however, is the creation of a channel which may trap soiling material, and prevent effective decontamination of the face shield for reuse. A further challenge observed with such incomplete depth cuts when prototyped was a higher incidence of brittle failure at or near the creases when folded. Our hypothesis is that the very small radius of curvature of the folding hinge created at this type of crease results in high localised stress concentrations, increasing the risk of a brittle failure. A further challenge with the above approach may be controlling the precise depth of the partial depth cut using a laser cutter, particularly when the sheet material may not rest completely flat on the laser cutting bed, or when precise laser power and speed settings may be difficult to reliably control.

#### 6.3.4 Full depth cut creasing

A further creasing method explored as part of this work was the use of complete depth cuts along the desired folding curve which do not completely join (for example “dashed” cuts), leaving sheet material in place at least in some locations across the creasing curve. A large body of literature has explored complete depth cut patterns for the creation of hinges in sheet materials [41–44].

While such a creasing approach would benefit from straightforward manufacture using a laser-cutter (at an estimated 30-90 seconds per shield) or potentially high-volume die-cutting, a key disadvantage of such creases is their permeability to fluids and aerosols. Furthermore, the gaps produced by such cuts could trap soiling material, and prevent effective decontamination for reuse. This work also explored the use of a flexible transparent fluid-repellent adhesive-backed sheet (contact paper) applied over complete depth cuts as a means of providing a fluid and aerosol barrier there. While such a method could help to provide protection from fluids and aerosols in designs using complete-depth cut creases, the robustness of such a laminated hinge when subject to multiple rounds of repeated folding and unfolding, in addition to decontamination involving liquid and manual abrasion, could be poor, potentially resulting in loss of protective function after a small number of reuses.

### 6.4 Folding

For all of the shields produced as part of this study, sheets were manually folded along creases. This folding procedure typically took between 30 and 90 seconds per shield, depending on desired fidelity of the fold beyond that required to satisfy performance requirements.

It should be noted that it is possible for users to potentially produce low quality shields when manually folding, if folds are made away from the creases (introducing local weaknesses and affecting worn shield geometry). When folded with reasonable care, however, excellent fold robustness and consistent worn geometry are achievable. This experience suggests that folding could be performed by first-time users of face shields upon opening of shield packaging, thus allowing for space-efficient transportation of shields in their flat-packed configuration.

### 6.5 Strap threading and attachment

Strap attachment clip folding, threading, and attachment to visors for all shields produced as part of this study was performed manually. Threaded straps could be shipped attached to flat face shields, resulting in worst-case packing thicknesses of approximately 5.0 mm, or unthreaded, requiring that users thread and attach these upon opening shield packaging, resulting in worst-case packing thicknesses approaching sheet material thickness at 0.5-1.0 mm. Strap threading and attachment could be performed in 1-3 minutes per shield. Apart from easily avoidable errors in threading pattern, strap attachment orientation, and tightness, high quality strap threading and attachment may easily be achieved by first-time users when provided with instructions.

### 6.6 Manufacturing method and material compatibility

It is of interest to identify which of the manufacturing methods discussed above are compatible with each other, so that producers of face shields may make best use of the manufacturing methods available to them. Manual cutting methods are generally likely to be incompatible with mechanised creasing methods, because such mechanised methods are likely to require precise boundary curves for registration, which may be difficult to achieve using some manual cutting methods. Laser and die-cutting methods are likely to be compatible with any creasing method. All methods considered are compatible with manual folding and strap threading and attachment, the only folding and strap attachment methods considered in this paper. Both PET and acetate are potentially compatible with all methods, however, as previously mentioned, brittle failures were observed in thin (250 micron) PET when produced using laser cutting and mechanical pressure creasing. Those manufacturing curved crease origami face shields are responsible for testing these according to medical device performance standards in their jurisdiction to ensure that the particular manufacturing methods and materials used in their production result in face shields which are fit for purpose.

## 7 Performance of curved-crease origami face shields

### 7.1 Performance assessment

A version of the face shield design presented in this paper (denoted model number HS-00-00-01) passed all of the tests described in the BSI’s PPE Technical Specification 2020/403 for Healthcare Professionals during the COVID-19 Pandemic, as tested on the adult reference head-form for eye protection evaluation described in EN 166, clause 17. The design tested used 510 micron PET, laser cutting, mechanical pressure creasing, and manual folding and strap attachment. The tests and checks performed are listed in Section 2.1.

This testing was part of the conformity assessment (CE marking approval) process required for permission from the United Kingdom Office for Product Safety and Standards to manufacture and distribute face shields for infection control during the COVID-19 pandemic. This process, which also involved a review of technical documentation and proposed manufacturing procedures, was granted for the manufacturing of this face shield design at the University of Cambridge Department of Architecture. This approval means that, according to UK public health guidance and European Standards, the design was deemed fit for purpose as a face shield for infection control during the COVID-19 pandemic, and subject to controlled manufacturing procedures, could be distributed as a face shield for infection control within the UK. It is important to note that this approval applies only to shields manufactured by University of Cambridge Department of Architecture using the materials and manufacturing methods associated with the shields which were tested as part of the approval process.

Preliminary feedback regarding shield designs was also obtained from a small number of healthcare workers. Comments focussed on the fit, comfort, degree of fogging, and overall size of the shields. Users reported that the shields were comfortable to wear for extended periods, and fogging was not reported as a concern. In hot conditions, sweating in the forehead rest region was identified as a potential issue, but was ultimately not found to be a significant concern. Early feedback from users also informed the design of the front visor, whose size in later design iterations was limited to prevent restriction of head movement.

A comprehensive study involving human participants to evaluate the performance of the face shield was outside of the scope of this work, and not required for CE certification. As this work did not involve randomising participants to different groups, did not involve any change to treatment or standards of care, and was not intended to provide generalisable research findings, it was deemed to fall outside the the scope of research as considered by the UK National Health Service (NHS) Health Research Authority [45] and no formal review by a Research Ethics Committee deemed necessary.

It is also important to note that, although preliminary user feedback did not report fogging as a concern, some design variants tested by the authors did exhibit some fogging. Increasing the space between the front visor and the wearer’s face and limiting excessive extension of the bottom of the front visor were identified as potential strategies to mitigate fogging if this appears to be a concern in future design variants. The above-mentioned BSI testing procedure for CE approval face shields for infection control in the COVID-19 pandemic did not include any tests for fogging.

### 7.2 Reusability evaluation

#### 7.2.1 Decontamination procedure

Further tests were conducted to assess the likelihood of the ability for the face shield presented in this paper to be safely reused. A preliminary evaluation of a potential decontamination procedure was conducted, whereby the strap was removed from the shield, the shield was flattened, and liquid soap was used to manually clean and disinfect the shield. Once dry, sterilising wipes containing chlorhexidine were used to sterilise the shield. Disinfection using sterilising wipes has been suggested in United States Centers for Disease Control and Prevention (US CDC) guidance [46] and in recent work using a similar face shield design [27] to likely be an effective means of decontaminating face shields for reuse in the context of the COVID-19 pandemic. As preparation for subsequent reuse, the shield was folded back into its three-dimensional configuration and a new strap was attached. This decontamination procedure appeared likely to satisfactorily remove all visible soiling, and assuming that the sterilising agent in the wipes was able to make complete contact with all portions of the plastic surface, is likely to be effective for inactivation of infectious agents. A complete experimental assessment of the efficacy of the decontamination procedure with regards to the inactivation of infectious agents is outside of the scope of this study. However, the use of sterilising wipes for decontamination of a similar face shield in a recent study [27], and the recommendation in US CDC guidance that sterilising wipes be used for reprocessing of face shields for reuse [46] suggest that decontamination using sterilising wipes is likely to be effective. [27] further demonstrated the use of isopropanol solution, ionised hydrogen peroxide, and ultraviolet light sterilisation for their similar face shield design. Such decontamination methods could also likely be applied to the face shield design presented in this paper. The manual decontamination procedure described above could be completed within 5 minutes for a single shield, but likely could be adapted for the rapid bulk decontamination of larger numbers of shields.

Subject to the development of an acceptable decontamination procedure for these, straps and clips could also be decontaminated and reused. Collaborative work by the authors has explored variants of the design presented in this paper which incorporate a headband consisting of a plastic strap of the same sheet material, possibly contiguous with the face shield sheet, which could be easily decontaminated alongside the face shield using the procedure described above, eliminating any face shield material waste as a result of decontamination.

#### 7.2.2 Fatigue testing

To assess the mechanical performance of the face shield subject to multiple reuses, a preliminary fatigue test was performed. The fatigue performance of a face shield using 510 micron PET, produced using laser cutting, mechanised pressure creasing, and manual strap attachment and folding was assessed. In this experiment, the face shield, with its strap removed, was folded from its three-dimensional configuration to its flat configuration repeatedly to assess the mechanical effects of repeated folding and unfolding, as would occur over multiple decontamination cycles. The shield was flattened and unflattened manually, and a distributed weight was applied over the entire shield surface to improve consistency of flatness with each cycle. To ensure consistency of folding into the three-dimensional configuration, the shield was folded to a target edge-to-edge distance *b* of 210±20 millimetres in its three-dimensional configuration.

No visually discernible damage to the creases occurred over 100 fold cycles, and no cracks in the shield were observed, meaning that fluid-impermeable barrier function of the shield was not compromised. A slight apparent reduction in folding stiffness was perceived after 5-10 fold cycles. These preliminary results suggest that mechanical failure is unlikely to occur in curved crease origami face shields using the materials and manufacturing methods tested, for a relatively high number of estimated reuses.

It should be noted that preliminary experiments on various materials and creasing methods throughout the prototyping process suggested a strong influence of material choice and creasing technique on the number of fold cycles before failure, and thus those adapting this design should ensure that the materials and creasing methods selected provide the desired crease robustness for the intended application. The effect of disinfecting agents on the optical and mechanical characteristics of the face shields was not evaluated.

## 8 Conclusions

This paper has identified the potential for curved-crease origami techniques to be used to efficiently produce large volumes of face shields for infection control using a wide range of manufacturing methods as appropriate to specific contexts.

Through an analysis of the state of the art in face shield design, this paper has highlighted the importance of rapid high-volume manufacture, the ability to be safely reused, resilience against disruptions to supply chain, manufacturing, and labour, and ease of replication and adaptation in the design of face shields for infection control. The design presented may be manufactured at high production rates, transported and stored efficiently. Subject to the validation of an approved decontamination procedure, the design could potentially be easily decontaminated and reused. It uses small quantities of inexpensive raw materials, requires only two unique materials and is likely to be easily produced using alternative raw materials. The design also has a simple geometry which may be easily replicated and adapted using manual or widely available digital fabrication methods.

In addition to reporting on the specific design developed in this work, this paper has also detailed the iterative physical-digital prototyping procedure used to develop the design, which may be used by others to further improve and adapt the design to new contexts and applications. The elastica curved crease parametric modelling approach presented allows for the rapid and accurate visualisation of predicted final forms of face shields in their worn configuration as determined by a small number of input parameters which may be controlled by the designer. This design and visualisation tool, implemented in Rhinoceros CAD software using the Grasshopper parametric modelling environment is available open-source in the supplemental data associated with this paper. Equivalent tools could easily be implemented in other software as needed, using the method described in this paper.

## 9 Future applications and potential of curved crease origami

Beyond their application specifically for face shields, the explorations conducted in this study have highlighted the potential for curved-crease origami techniques to be applied in the design of other PPE and wearable devices. Curved-crease origami forms consist of curved surfaces which may conform well to human body forms, providing flexible contact regions which distribute attachment forces across a large area, reducing pressure which might otherwise cause discomfort or even soft-tissue injury. Wearables may also benefit from the compliant mechanism behaviour of curved-crease origami forms, as in the case of the face shield design presented, whereby the process of donning the shield reconfigures the shield from its flat to its three-dimensional form. Furthermore, as with other origami techniques, curved-crease origami allows for cost-effective and straightforward manufacture from sheet materials.

The severity and urgency of the COVID-19 pandemic have resulted in remarkable innovations from the design community, revealing extraordinary opportunities for improvements in the quality, efficiency, and sustainability of provision of critical goods and services globally, particularly to the most vulnerable. We are encouraged by the potential for further focused, collaborative, and open-source design in the field of folded structures to help address the numerous challenges facing humanity now and in the future.
